# The Central Conserved Region (CCR) of Respiratory Syncytial Virus (RSV) G Protein Modulates Host miRNA Expression and Alters the Cellular Response to Infection

**DOI:** 10.3390/vaccines5030016

**Published:** 2017-07-03

**Authors:** Abhijeet A. Bakre, Jennifer L. Harcourt, Lia M. Haynes, Larry J. Anderson, Ralph A. Tripp

**Affiliations:** 1 Department of Infectious Diseases, College of Veterinary Medicine, University of Georgia, Athens, GA 30602, USA; bakre@uga.edu; 2 National Center for Immunization and Respiratory Diseases, Centers for Disease Control and Prevention (CDC), Atlanta, GA 30333, USA; zaq6@cdc.gov (J.L.H.); loh5@cdc.gov (J.L.H.); 3 Division of Pediatric Infectious Diseases, Emory University Atlanta, Atlanta, GA 30322, USA; larry.anderson@emory.edu

**Keywords:** respiratory syncytial virus, RSV, microRNAs, CX3CR1, IFNλ

## Abstract

Respiratory Syncytial Virus (RSV) infects respiratory epithelial cells and deregulates host gene expression by many mechanisms including expression of RSV G protein (RSV G). RSV G protein encodes a central conserved region (CCR) containing a CX3C motif that functions as a fractalkine mimic. Disruption of the CX3C motif (a.a. 182–186) located in the CCR of the G protein has been shown to affect G protein function in vitro and the severity of RSV disease pathogenesis in vivo. We show that infection of polarized Calu3 respiratory cells with recombinant RSV having point mutations in Cys173 and 176 (C173/176S) (rA2-GC12), or Cys186 (C186S) (rA2-GC4) is associated with a decline in the integrity of polarized Calu-3 cultures and decreased virus production. This is accompanied with downregulation of miRNAs let-7f and miR-24 and upregulation of interferon lambda (IFNλ), a primary antiviral cytokine for RSV in rA2-GC12/rA2-GC4 infected cells. These results suggest that residues in the cysteine noose region of RSV G protein can modulate IFN λ expression accompanied by downregulation of miRNAs, and are important for RSV G protein function and targeting.

## 1. Introduction

Respiratory syncytial virus (RSV) causes serious lower respiratory tract illness in the young and the elderly leading to substantial morbidity and hospitalizations [1,2,3,4,5,6]. There is no safe and effective vaccine to prevent RSV. Previous vaccination approaches using formalin-inactivated RSV (FI-RSV) were associated with an increased risk of serious RSV illness upon subsequent natural infection [7,8]. Vaccine strategies have focused on using either live-attenuated viruses, inactivated whole virus vaccines [9] or subunit vaccines targeting the two major surface viral proteins, RSV fusion (F) and/or attachment (G) proteins [10,11,12]. Insufficient or over-attenuation, induction of enhanced disease with natural infection, or inadequate protection against infection [13,14,15,16] have blocked moving these vaccines to licensure and challenged the understanding of events at the host–virus interface.

The G protein is the attachment protein of RSV, and is expressed as both a membrane-bound and a secretory form [17] via alternative translation initiation and proteolytic splicing [18]. RSV G protein is organized as an N-terminal cytosolic region, a transmembrane region, and a variable ectodomain consisting of two mucin-like domains separated by a non-glycosylated central conserved region (CCR). The CCR contains four cysteine residues that form a cysteine noose, formed by disulfide bonds between a.a. 173–186 and 176–182 [19] and a CX3C chemokine motif (a.a. 182–186) that mimics fractalkine (FKN) [20]. RSV G protein CCR interacts with CX3CR1, recently identified as a RSV receptor [21,22] The molecular mimicry and interaction between the CX3C motif and CX3CR1 results in G protein-induced lymphocyte chemotaxis that can be inhibited by anti- G antibody [20]. Disrupting the CX3C-CX3CR1 interaction reduces TNFα and type I interferon (IFN) production in vitro [23] and alters CX3CR1+ cell trafficking, cytokine, chemokine and substance P expression [24,25]. Blocking the CX3C-CX3CR1 interaction also reduces viral load, bronchoalveolar lavage (BAL) leukocyte infiltration [26], immune cell trafficking, and IFNα and interleukin-4 (IL-4) levels in BAL of CX3CR1-deficient mice infected with RSV [27]. Anti-RSV G prophylaxis or treatment reduces pulmonary mucus production, inhibits infection-induced airway dysfunction and reduces pulmonary cell infiltration in response to RSV infection [23,26,28]. Thus, RSV G protein, and the G protein CX3C motif, has a key role in immunity and disease pathogenesis. Since the G protein CX3C motif is significant to infection of primary human airway epithelial cells [21,28,29], the contribution of the RSV G protein to disease is more remarkable than that otherwise appreciated. The RSV G protein is a valuable target for RSV vaccine development [21,24,26,30,31,32] and understanding the role of the CX3C motif in both innate and adaptive immune responses is important to developing RSV G protein-based vaccines.

To understand the role of the G protein CX3C motif and host gene expression, a human airway epithelial cell line, Calu-3 was examined. Calu-3 cells recapitulate the response of proximal airway epithelial cells to a variety of viral pathogens [33,34,35,36,37]. Calu-3 cells polarize into apical and basolateral surfaces in trans-well culture systems, but do not differentiate into multiple cell types in contrast to cultured normal human bronchoepithelial (NHBE) cells [38]. Polarized, liquid-covered cultured Calu-3 cells are susceptible to RSV infection, and support viral transcription and translation followed by apical progeny virus release beginning 3–4 weeks post-infection (pi) resulting in declining polarization [39]. RSV infection of Calu-3 cells causes minimal cytopathology or alterations in tight junction formation [39], which is consistent with cultured NHBE systems, and mouse and human studies [39,40,41,42,43,44,45,46]. In this study, Calu-3 cells were infected with either wild-type RSV/A2, or recombinant viruses containing mutations of the cysteines in the CCR of RSV G, (rA2-GC12, C173/176S, or rA2-GC4, C186S). Polarization status, viral replication, cytokine, chemokine mRNA production and microRNA (miRNA) expression were monitored following infection. The data show that perturbing the G protein CX3C motif modulates viral replication, cytokine and chemokine mRNA expression profiles and these correspond to deregulated miRNA expression during infection. These studies also show that miRNA expression is regulated by the organization of the central CCR domain in the RSV G protein, which may determine the pattern of host gene expression in response to infection.

## 2. Materials and Methods

### 2.1. Cell Lines

Vero cells (karyotyped at the CDC Atlanta, GA, USA) were maintained in Dulbecco’s Modified Essential Medium (DMEM, Life Technologies, Grand Island, NY, USA) with 10% heat-inactivated fetal bovine serum (DMEM-10%, FBS, HyClone, ThermoFisher Scientific, Rockford, IL, USA), at 37 °C and 5% CO_2_. Calu-3 cells were maintained in monolayer cultures in T-75 cm^2^ flasks in Eagle’s Minimum Essential Medium (EMEM, Life Technologies) supplemented with 20% heat-inactivated FBS, 0.1 mM non-essential amino acids (Life Technologies), 2 mM L-glutamine (Life Technologies), and 10 mM HEPES (Invitrogen, Carlsbad, CA, USA) EMEM-20% +S). Calu-3 cells were cultured as previously described [39,47]. Briefly, Calu-3 cells were seeded at a density of 6.0 × 10^5^ cells/cm^2^ onto polyester Transwell inserts (0.33 cm) with 0.3μ pores (Corning, Lowell, MA, USA) in EMEM supplemented with 10% FBS (EMEM-10%).

### 2.2. Measurement of TEER

Medium was changed every 3–4 days and polarization was monitored by determining trans-epithelial electrical resistance (TEER) of each Transwell culture, using an EVOM voltohmmeter (World Precision Instruments, Sarasota, FL, USA) and STX2 electrodes [47].

### 2.3. Viruses and Infections

Recombinant viruses rA2-GC12 and rA2-GC4 were generated by co-transfecting HEp-2 cells (ATCC-CCL23) with plasmids encoding T7 RNA polymerase promoter driven F, N, P, L, and M2-1 proteins of RSV strain A2, and a plasmid encoding the RSV G protein mutant antigenome cDNA, followed by infection with a modified vaccinia Ankara virus (MVA) expressing the T7 RNA polymerase [48]. Recombinant RSV (rA2) were amplified from the supernatant of transfected cells by serial passage on Vero cells. rA2-GC12 contains Cys to Ser point mutations at a.a. 173 and 176 while rA2-GC4 contains a Cys to Ser point mutation at a.a. 186. The rA2-GC12 mutant is thus a CCR variant while rA2-GC4 mutant is a true CX3C mutant. Sequence analysis of the RSV F gene revealed changes in 2 amino acids between RSV A2 and the rA2 recombinant viruses, K66E and Q101P [49]. The observed mutations are located in variable regions of the F protein within the A strain of RSV [50]. These strains (rA2-GC12 and rA2-GC4) were propagated in Vero cells as described [48], and determined to be mycoplasma free. Titered virus stocks at multiplicity of infection (MOI) = 1.0 were adsorbed to polarized Calu-3 cell monolayers grown on trans-well inserts (exhibiting a TEER ≥ 1000 Ω × cm^2^) for 2 h at 37 °C in serum-free EMEM followed by removal of inoculum and replacement with complete EMEM containing serum [39] and incubated for indicated time points. Mock infections were carried out using equal volume of Vero cell lysates for respective time points. Media from apical and basolateral chambers were removed at designated time points and replaced with fresh media. Removed media from two pooled wells were used in plaque assays to determine viral titers in apical supernatants. RSV infection of Calu-3 cells does not produce syncytia, so viral titers in culture supernatants were determined by a plaque assay on Vero cell monolayers as described [39,47].

### 2.4. RNA Isolation and qRT-PCR

Following two washes of both the apical and basolateral surfaces of polarized Calu-3 cells with sterile Dulbecco’s phosphate buffered saline (D-PBS), total cellular RNA was extracted from Calu-3 cells using PureLink^®^ RNA purification kit (Invitrogen) per the manufacturer’s protocol, and stored at −20 °C until use. Expression of the RSV matrix (M) gene was determined using primers (forward, position 3257-3282) 5′-GGC AAA TAT GGA AAC ATA GCT GAA-3′ and (reverse, position 3312-3340) 5′-TCT TTT TCT AGG ACA TTG TAY TGA ACA G-3′) [51]. Briefly, real-time quantitative RT-PCR was performed on a Stratagene Mx3000 detection system (Agilent Technologies, Santa Clara, CA, USA). Threshold cycles (*C_t_*) for each sample were calculated and serial dilutions of known PFUs of RSV A2 RNA were used to obtain a standard curve, from which the relative amount of genome, in PFU/mL, was determined for each sample. Commercially available TaqMan probe mixes were used to determine IFNα1, IFNλ, suppressor of cytokine signaling (SOCS-1) and SOCS3 expression profiles relative to 18S rRNA (Applied Biosystems, Foster City, CA, USA).

### 2.5. Plaque Assays

Viral titers in the apical supernatants of infected cells were determined as described previously [52]. Briefly, 10-fold serial dilutions of cell supernatants, prepared in serum free DMEM (SF-DMEM), were added to confluent Vero cell monolayers in 96-well plates. After adsorption for 2 h at 37 °C, cell monolayers were overlaid with DMEM containing 10% FBS and 0.5% methylcellulose (Sigma, St. Louis, MO, USA), incubated at 37 °C for 3 days, and plaques enumerated by immunostaining with murine monoclonal antibodies against RSV G protein and RSV F proteins (clones 131-2G and 131-2A, respectively).

### 2.6. MicroRNA Analysis

Total RNA from Calu-3 cells mock-infected and cells infected with RSV A2, rA2-GC12 or rA2-GC4 viruses for various time points was isolated using PureLink^®^ RNA purification kit (Invitrogen) per the manufacturer’s protocol, and stored at −20 °C until use. Total RNA was quantified using NanoDrop 1000 spectrophotometer and DNAse treated. Equal amount of DNA free RNA (1 μg) was polyadenylated and reverse transcribed using High sensitivity miRNA cDNA synthesis kit (Agilent, USA). First strand cDNA was diluted as appropriate and used for miRNA amplification in a Stratagene Mx3000/3005P real time PCR machine. In all amplifications, forward oligo used was miRNA specific while reverse primer was complementary to an adaptor added during reverse transcription. In addition, 18S rRNA was used as a housekeeping gene in all experiments. All quantitative experiments follow minimum information for quantitative experiments (MIQE) guidelines [53].

### 2.7. High Content Microscopy

Calu-3 cells were plated overnight in 96-well plates and transfected with inhibitor negative control (INC), mimic negative control (MNC), or miR-24 specific inhibitor/mimic using Dharmafect 1 (DF1) for 48 h. Transfections were validated in parallel using a Dy547-labelled miRNA transfection and >90% cells were transfected. Transfected cells were fixed with 4% Formalin in PBS and then blocked overnight in 5% Blotto in PBS. Cells were stained for CX3CR1 expression using rabbit anti-CX3CR1 antibodies labelled with allophycocyanin (APC). Nuclei were counterstained with Hoechst 33342 (1 μg/mL) for 10 min followed by a PBS wash. Plates were analyzed using an ArrayScan VTI high content microscope (ThermoFisher Scientific). The percentage of CX3CR1 positive cells was determined after counting 20,000 independent events in each treatment in triplicate.

### 2.8. Statistical Analysis

All experiments were individually performed *n* > 3, and representative data are presented. Statistical analyses were performed using one-way or two way ANOVA analysis between data values for mock or RSV-A2-infected and rA2-GC12 or rA2-GC4-infected samples using GraphPad Prism ver 5.0 (GraphPad Software, La Jolla, CA, USA). Differences were considered significant at *p* ≤ 0.05.

## 3. Results

### 3.1. RSV G Protein CCR Modifies Polarization of Calu-3 Cells

Calu-3 cells, derived from a human bronchus carcinoma, are of mixed phenotype [36] and gene expression profiles of Calu-3 cells grown at air-liquid interface (ALI) are similar to primary lung epithelial cells grown at ALI making the cells a useful model for in vitro studies [54]. Our preliminary observations suggest that between 15–55% of Calu-3 cells express the fractalkine receptor, CX3CR1 (unpublished data). Polarized Calu-3 cells at ALI were infected with RSV/A2, rA2-GC12 and rA2-GC4 (MOI = 1.0). rA2-GC12 and rA2-GC4 viruses have the RSV/A2 backbones but differ in the sequence of the CCR (Table 1).

**Figure 1:**
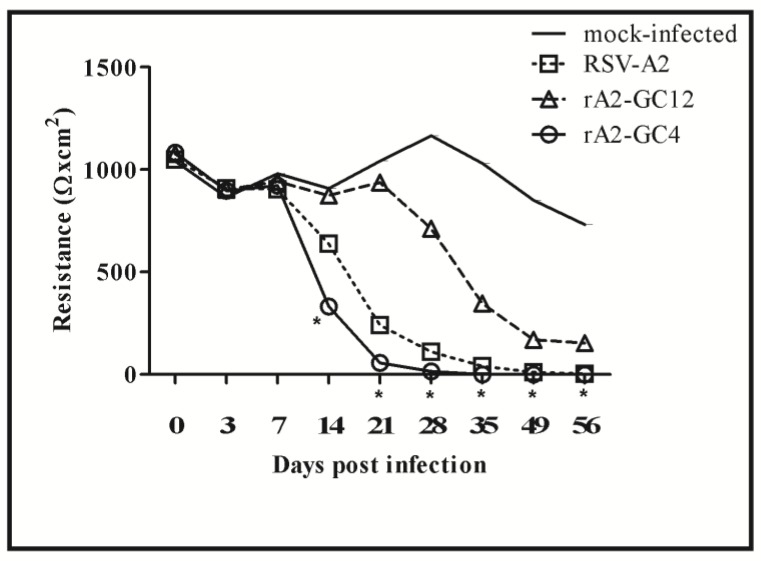
rA2-GC4 infection results in a decline of polarized Calu-3 cells. Data represent the median resistance measurement, Ω × cm^2^, of polarized liquid-covered culture (LCC) Calu-3 cells infected apically with RSVA2, rA2-GC12 or rA2-GC4 at a multiplicity of infection (MOI) of 1 for varying time points. The data presented is representative of one of three independent experiments. * represents significance (*p* ≤ 0.05) relative to mock as determined by one-way ANOVA and Bonferroni post hoc comparison of all data at α = 0.05).

Since the RSV G protein is involved in attachment of the virus to the cell surface via glycosaminoglycans [55] and CX3CR1 [21], differences in infectivity of RSV A2 vs rA2-GC12 and rA2-GC4 viruses were determined using trans-epithelial electrical resistance (TEER) over a period of 56 days (Figure 1). RSV A2 infection is characterized by loss of TEER in primary bronchial epithelium and was used as a surrogate marker of infectivity [56].

RSV/A2 infection (MOI = 1.0) significantly (*p* = 0.00017) reduced TEER to <1000 Ω × cm^2^ within 14 days post-infection (pi), in contrast to mock-infected cells, which retained TEER >1000 Ω × cm^2^ up to 35 days pi. rA2-GC4 infection accelerated the loss of TEER relative to both mock or RSV/A2 infected cells, with a significant loss of TEER by 14 days pi (*p* = 0.035). Loss of TEER was not as pronounced for rA2-GC12 infected cells relative to mock until 28 days pi and remained higher than RSV A2 infected cells through 56 days pi. These data suggest that the organization of the CCR influences RSV infectivity and the degree of pathogenicity or virulence.

**Figure 2:**
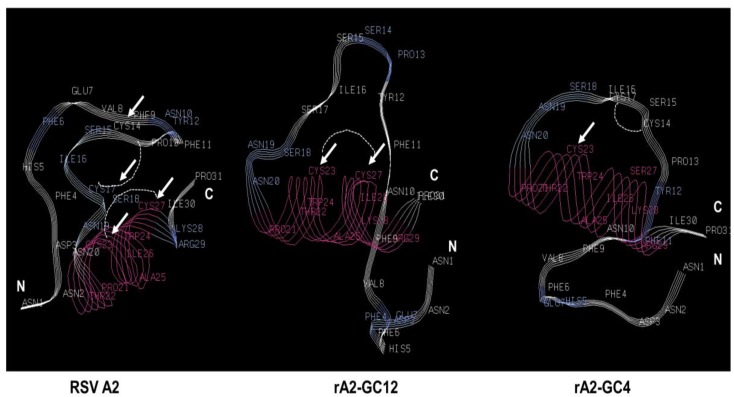
Mutations in the CX3C motif affect folding patterns of the cysteine noose. Peptide sequences corresponding to the RSV G protein central conserved region (CCR) were analyzed for folding pattern using Pepfold [57]. Arrows indicate positions of cysteine in the RSV A2 backbone and the changes in the rA2-GC12 and rA2-GC4 peptides.

Ab initio secondary structural analysis of the RSV A2, rA2-GC12 and rA2-GC4 CCR using the Pepfold de novo peptide structure prediction server [57] showed that the RSV A2 and rA2-GC12 peptides are folded similarly. Folding patterns of the rA2-GC4 CCR peptide, however, were appreciably different (Figure 2), which could alter the binding kinetics as well as the ligands this motif interacts with during infection and partly explain the differential cytokine and miRNA expression profiles. It is possible that disruption of the CX3C motif in rA2-GC4 disrupts CX3CR1-CX3CL1 interaction, and deregulates downstream cytokine signaling.

### 3.2. RSV G Protein CCR Modulates RSV Replication

**Figure 3:**
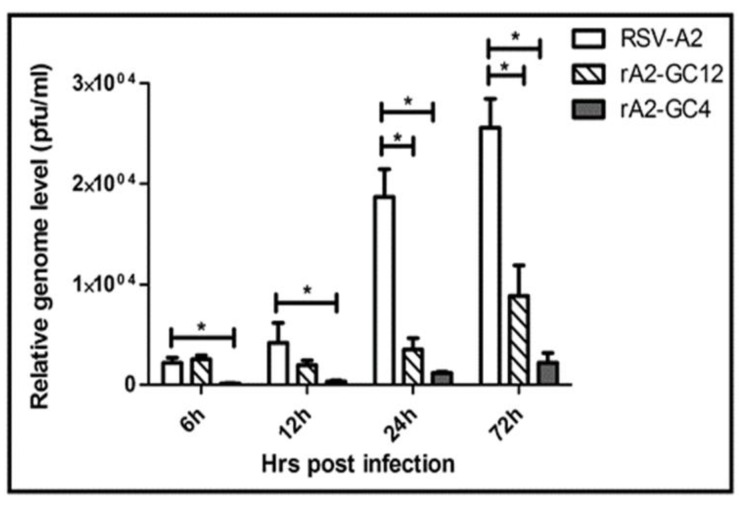
rA2-GC4 and rA2-GC12 viruses replicate poorly in polarized liquid covered culture (LCC)-Calu3 cells. Data represent relative levels of RSV M mRNA from RSV/A2, rA2-GC12 or rA2-GC4 virus infected LCC-Calu3 wells (*n* = 3) ± SEM in PFU/mL as determined by qRT-PCR at indicated time points. * indicates statistical significance (*p* ≤ 0.05) as determined by one-way ANOVA followed by the post hoc Bonferroni test at a significance level (α = 0.05).

To determine whether the mutations in the G protein CCR of the recombinant viruses affected virus replication, Calu-3 cells were infected with RSV-A2, rA2-GC12 or rA2-GC4 (MOI =1.0), and the expression of intracellular RSV M mRNA was determined at 6 h, 12 h, 24 h pi RSV A2 infection. RSV M expression was reduced following rA2-GC12 and rA2-GC4 infections (Figure 3) at all time points. The reduction in RSV M gene expression following rA2-GC12 infection was significant (*p* < 0.05) at 24 h and 72 h pi, whereas the reduction in RSV M gene expression in rA2-GC4 infected cells was significant (*p* < 0.05) at all time points examined. These data suggest that rA2-GC12 and rA2-GC4 viruses are able to infect, but unable to replicate, as efficiently as the parent A2 strain. Differences in apical viral titers from RSV A2, rA2-GC12 or rA2-GC4 infections, ranging between the lower limit of detection (20 PFU/mL) and 2.5 × 10^3^ PFU/mL for each infection, were not significant at day 3 pi. No changes in the sequence of the RSV G gene were observed in progeny virus produced even at 35 days pi, suggesting that the mutant G protein gene is stable during prolonged infection of Calu-3 cells.

### 3.3. G Protein CCR Influences the Cell Cytokine and Chemokine Response to RSV Infection

**Figure 4:**
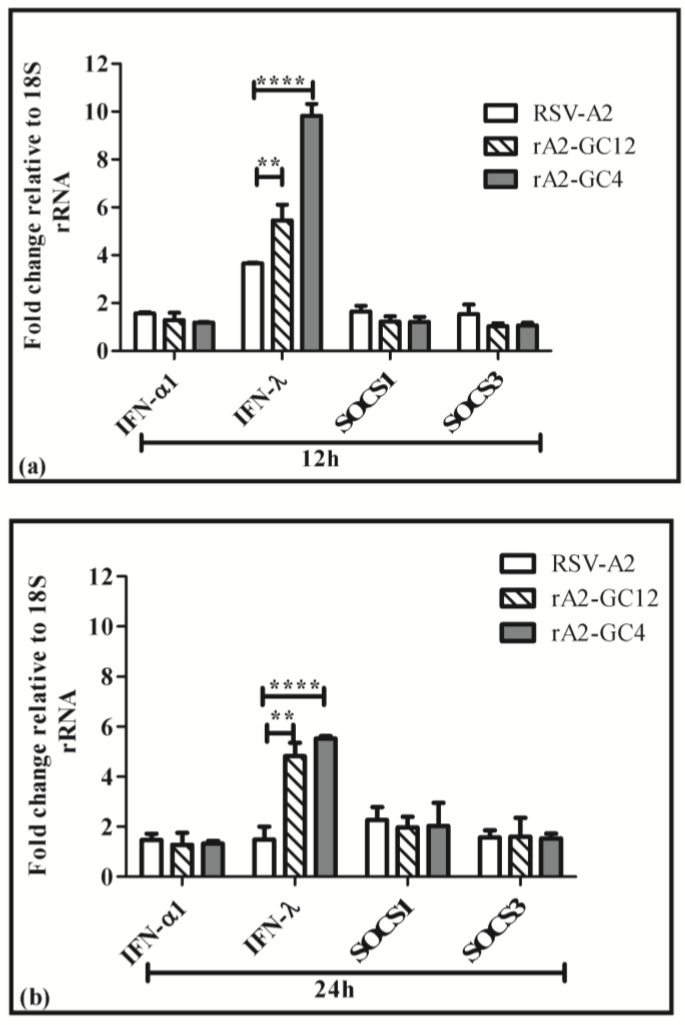
The central conserved region (CCR) of RSV G protein alters IFNλ expression. Polarized LCC Calu-3 cells were infected with RSV/A2, rA2-GC12 or rA2-GC4 virus (MOI = 1.0). Total RNA was isolated from mock and infected cells at 12 h and 24 h pi. Expression of IFNα1, IFN λ, SOCS-1 and SOC-3 were determined by gene specific primer-probe combinations using a one-step qRT-PCR assay at 12 h (**a**) or 24 h (**b**) pi Data represent ± SEM of three independent experiments. ** indicates statistical significance (*p* < 0.01) and **** indicates statistical significance (*p* < 0.001) as determined by two-way ANOVA and the Bonferroni post hoc test.

A Cys to Ala mutation in the RSV G protein has been shown to induce IFNλ [23], which could cause the reduction in RSV M expression in response to infection with rA2-GC4 and rA2-GC12 viruses. IFN λ and SOCS-1 and SOCS-3 expression were evaluated at 12 h and 24 h following mock, RSV A2, rA2-GC12 and rA2-GC4 infections. IFNλ secretion was significantly (*p* < 0.01) upregulated in rA2-GC12 and rA2-GC4 infected cells relative to RSV A2 after normalizing to mock, while expression of SOCS-1 and SOCS-3 was not affected (Figure 4a,b). We previously demonstrated that RSV6340 (a wild type virus) infection could induce SOCS-1/-3 and IFN expression, an effect that was severely ablated upon infection with a recombinant virus lacking the G protein (RSV ΔG) [58]. UV inactivated RSV induced SOCS-3 but not SOCS-1 and failed to induce an IFN response. Since CX3CR1 has been recently identified as a RSV co-receptor [21,22,29], this suggests that the RSV G CX3C-CX3CR1 interaction induces SOCS-1/-3. Ab initial predictions of both the rA2-GC12 and –GC4 CCR clearly show a different folding profile suggesting that these proteins may be unable to interact with surface CX3CR1, thus explaining the lack of SOCS-1/-3 induction in mutant virus infection and emphasizing the need for an intact CX3C motif for G protein-mediated immunosuppression.

### 3.4. Perturbing the CX3C Motif Deregulates Host miRNA Responses

**Figure 5:**
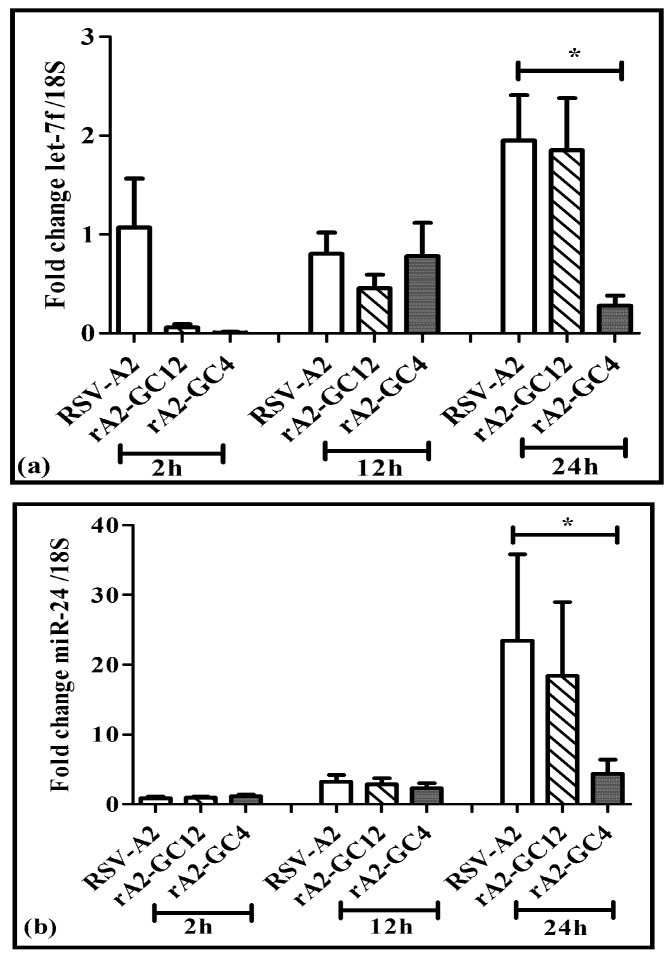
The central conserved region (CCR) of RSV G protein alters miRNA expression. Total RNA from polarized LCC Calu-3 cells mock infected, or infected apically with RSV A2, rA2-GC12 or rA2-GC4 at MOI = 1 was isolated using Trizol as per manufacturer’s protocol. Expression of let-7f (**a**) and miR-24 (**b**) was determined with RT-qPCR using a miRNA specific forward oligo and a universal oligo relative to 18S as housekeeping gene. Data represent ±SEM three independent experiments. * indicates *p* ≤ 0.05 relative to values from RSV-A2.

RSV is known to modulate host miRNA expression, an effect that influences cell cycle and immune response pathways [59]. Five miRNAs (let-7, miR-24, miR-26b, miR-337 and miR-520a) were upregulated early during RSV infection where RSV G protein primarily affected induction of miRNA let-7f [59]. Let-7f and miR-24 are the most abundant miRNAs induced in type II epithelial (A549) cells upon RSV infection [59]. Thus, overexpression of IFNλ observed in rA2-GC4 infected cells may be linked to the suppression of regulatory miRNAs, particularly miRNAs let-7f and miR-24-3p (referred to as miR-24 hereafter). Similar to the results in A549 cells, RSV A2 infection in Calu-3 cells also led to upregulated let-7f and miR-24 relative to mock at 24 h pi (Figure 5a,b). Infection of Calu-3 cells with rA2-GC12 virus also induced let-7f and miR-24 expression similar to RSV A2. In contrast, the expression of let-7f and miR-24 was markedly reduced in rA2-GC4 infected cells (Figure 5a,b) at 24 h. These data suggest that an intact CX3C motif is essential for RSV G protein driven let-7f and miR-24 induction.

## 4. Discussion

The cysteine noose region of the CX3C motif in the CCR of RSV G protein elicits protective CTL activity [60], and induces a protective anti-RSV G protein antibody response [32,61]. The CX3C motif in the RSV G protein CCR contributes to RSV disease pathogenesis via molecular mimicry with the CX3CR1 receptor and alters a number of immune responses to RSV infection [20,23,24,25,31,62,63]. Thus, one may alter the CCR as a strategy to evoke a strong protective anti-RSV response while minimizing the development of RSV-associated pathogenesis. To understand the impact of the RSV G protein CX3C motif on viral replication, IFN and miRNA expression were determined in Calu-3 cells infected with wildtype RSV A2, or rA2-GC12, or rA2-GC4 mutant viruses, which have mutations of the cysteines in the CX3C site of the G protein. RSV A2 and rA2-GC12 virus infection led to loss of polarization in Calu-3 cells, an effect that was considerably accelerated during rA2-GC4 infection, suggesting differential viral replication kinetics. However, the replication kinetics of both rA2-GC12 and rA2-GC4 viruses were substantially reduced relative to wildtype RSV A2. Since previous studies show that RSV G protein suppresses IFN induction of ISGs via enhanced expression of SOCS-1 and SOCS-3, and regulates miRNA expression during infection, the expression profiles of type I and III cytokines and microRNAs were examined; type III IFN and miRNAs let-7f and miR-24 were found to be altered between wildtype and RSV mutant virus infections. Type I IFN, SOCS-1 and SOCS-3 expression were not detectably different between RSV A2 and RSV mutant infection, but type III IFN secretion was increased in rA2-GC12 and GC4 infected Calu-3 cells. Since type III IFNs are the predominant cytokines expressed during RSV infection [64,65,66], these data suggest that altering the CX3C motif disconnects RSV G protein-mediated suppression of IFN expression, the induction of ISG expression, and establishment of an anti-viral state in infected cells. Peptide structure predictions showed that the CX3C domain from rA2-GC12 folded similarly to that of RSV A2, but that folding of the G protein CCR from rA2-GC4 was drastically altered due to disruption of the CX3C motif (Figure 2). This increase in IFN may explain the relatively lower viral replication kinetics of rA2-GC4. Since the RSV G protein has been shown to affect miRNA induction, particularly the expression of let-7f and miR-24 [59], miRNA profiles in RSV infected cells were also examined in RSV A2, rA2-GC12 and rA2-GC4 infections at 2 h, 12 h and 24 h pi. Expression of let-7f and miR-24 in rA2-GC12 and rA2-GC4 infected cells was not statistically (*p* < 0.05) different from RSV A2 infection at the 2 h and 12 h time-points but was significantly (*p* < 0.05) reduced in rA2-GC4 infected cells relative to RSV A2 infection at 24 h pi, suggesting that type III IFN induction is accompanied by reduced expression of miR-24 and let-7f expression. Since translation inhibition is the main mode of miRNA action, it is possible that reduced miR-24 expression increases IFN translation. Two distinct miR-24 loci in the human genome [67] are transcribed to produce the same mature miR-24. In addition to the regulation of IFNλ, miR-24 has important roles in cell cycle regulation. Inhibition of miR-24 deregulates cell cycle in A549 cells [68,69] and the rapid loss of polarization in rA2-GC4 infected cells may be due to inhibition of miR-24 and its targets.

We have previously shown that RSV NS1 also can suppress miR-24 expression via Tumor Growth Factor - beta (TGF-β) potentially to modulate cell cycle [70]. Together, these data suggest that an intact RSV G protein CX3C motif regulates cytokine and miRNA expression though the precise mechanisms that govern these interactions are not presently known. This is the first report showing that altering the CX3C motif can alter miRNA expression profiles and contribute to deregulated cytokine expression. A previous study in A549 cells showed that infection with CX4C RSV carrying a Cys to Ala mutation at position 186 (similar to rA2-GC4) in the CX3C motif induces expression of both type I and III interferons [23]. We did not observe any differences in type I IFN expression in our system, but did observe considerably less type III IFN induction, an effect that could be linked to differences in the cell lines used. We also observed that three other cytokines—MIP-1β, GCSF and IL-15—were induced in rA2-GC4 infected cells with concomitant decreases in miR-24 and let-7f miRNAs respectively, which are predicted to regulate these cytokines (Supplementary Materials Figure S1). These data suggest that the CX3C motif affects cytokine expression via miRNA induction.

RSV G protein is known to have multiple functions beyond acting as a viral attachment protein [20], which includes affecting Toll-like receptors (TLR4) [71,72] and TLR3 [73], induction of IL-1β and C-C Motif Chemokine Ligand 5 (CCL5) expression by NHBE cells [74], inducing suppressor of SOCS proteins [58,75] and suppression of type I interferon expression [75]. Although the RSV G protein contributes to development of enhanced respiratory disease following natural infection of FI-RSV vaccinated individuals, antibodies against RSV G protein CCR are protective and reduce disease severity [26,28,30,31,32,51]. However, the specific impact of the RSV G protein CCR, and specifically the CX3C motif on the host cellular response to RSV infection, is not well-characterized. In this study, rA2-GC4 infection of Calu-3 cells was associated with an accelerated decline in TEER in polarized Calu-3 cells relative to the other RSV mutant viruses. In contrast, infection with rA2-GC12, a recombinant virus containing Cys→Ser mutations in positions 173 and 176 of RSV G protein that is predicted to affect the formation of both disulfide bonds of the cysteine noose, but not disrupt the CX3C motif, is associated with a less severe, delayed decline in TEER in response to RSV infection. The differences in viral mRNA detected in Calu-3 cells at 15 min to 72 h pi with RSV-A2, rA2-GC12 and rA2-GC4 reveal that the differences in TEER do not correlate with differences in RSV entry or early replication in Calu-3 cells, and suggest that the differences in TEER and viral production following infection with the CCR mutant viruses are not the result of changes in the ability of the CX3C mutant viruses to interact with CX3CR1. However, it is possible that the mutations impact virus internalization, packaging or release, and further studies are needed to determine the precise mechanisms of these changes. It is also possible that the differences in TEER are due to differing numbers of Calu-3 cells being infected following infection of Calu-3 with RSV A2, rA2-GC12 and rA2-GC4, or to induction of apoptosis following rA2-GC4 infection. Recent studies suggest that the CX3C motif is important in infection, and it is possible that differences in RSV M transcripts between RSVA2 and rA2-GC4 and rA2-GC12 viruses could relate to different infectivity.

The exact mechanism by which the RSV G protein CCR mutations are associated with enhanced cytokine and chemokine expression is unknown and requires further investigation. It is expected that mutations in cysteine residues 173 and 176 (rA2-GC12), or in cysteine residue 186 (rA2-GC4), alter the ability of RSV G protein to form the disulfide bonds, thereby impacting the formation of the cysteine noose and ultimately the structure of RSV G protein. The RSV G protein CCR demonstrates structural homology to TNF-α receptor [76], and RSV G protein disrupts TLR3 and TLR4 mediated signaling [73]. It is thus possible that the mutations of the recombinant viruses disrupt the ability of RSV G protein to signal through these pathways. Furthermore, RSV G protein regulates the early expression of miRNAs after infection or treatment of cells with purified RSV G protein, including let-7 family members [59,77], and let-7 miRNA expression is associated with decreased expression of multiple genes that may impact the expression of various cytokines and chemokines, including SOCS-3 [59], although the mechanism by which RSV G protein mediates miRNA regulation is not defined. It is also possible that the RSV G protein CX3C motif regulates miRNA expression in response to infection. Sequence analysis of RSV F proteins in the mutant viruses showed two point mutations K66E and Q101P. The impact of these two mutations on the function of RSV G CX3C motif described in this manuscript is not understood and needs further exploration. Our data demonstrate that the RSV G protein CCR is important in modulating the host cellular response to RSV infection, affecting the production of infectious virus, cytokine and chemokine expression, and polarized culture integrity in response to infection. This study highlights the function of the RSV G protein CCR and CX3C motif in regulating the host cellular response to infection, and demonstrates the importance of considering the impact this motif has on the host cellular response to infection when designing RSV vaccines.

## 5. Conclusions

We conclude that the organization of the CX3C motif has an impact on regulation of host immune responses and, consequently, outcomes of RSV infection.

## Figures and Tables

**Table 1:** Sequences of the conserved central region of RSV G protein.

Virus	Sequence
	173 176 182 186
RSV-A2	NNDFHFEVFNFYPCSICSNNPTCWAICKRIP
rA2-GC12 (C173/176S)	NNDFHFEVFNFYPSSISSNNPTCWAICKRIP
rA2-GC4 (C186S)	NNDFHFEVFNFYPCSICSNNPTCWAISKRIP

## Supplementary Materials

The following are available online at www.mdpi.com/2076-393X/5/3/16/s1.
