# Supplementary material for: The Central Conserved Region (CCR) of Respiratory Syncytial Virus (RSV) G Protein Modulates Host miRNA Expression and Alters the Cellular Response to Infection
Source: Vaccines (Basel). 2017 Jul 3;5(3):16. doi: 10.3390/vaccines5030016 (PMC5620547; doi:10.3390/vaccines5030016)
Supplement: Supplementary file 1 [file vaccines-05-00016-s001.docx]

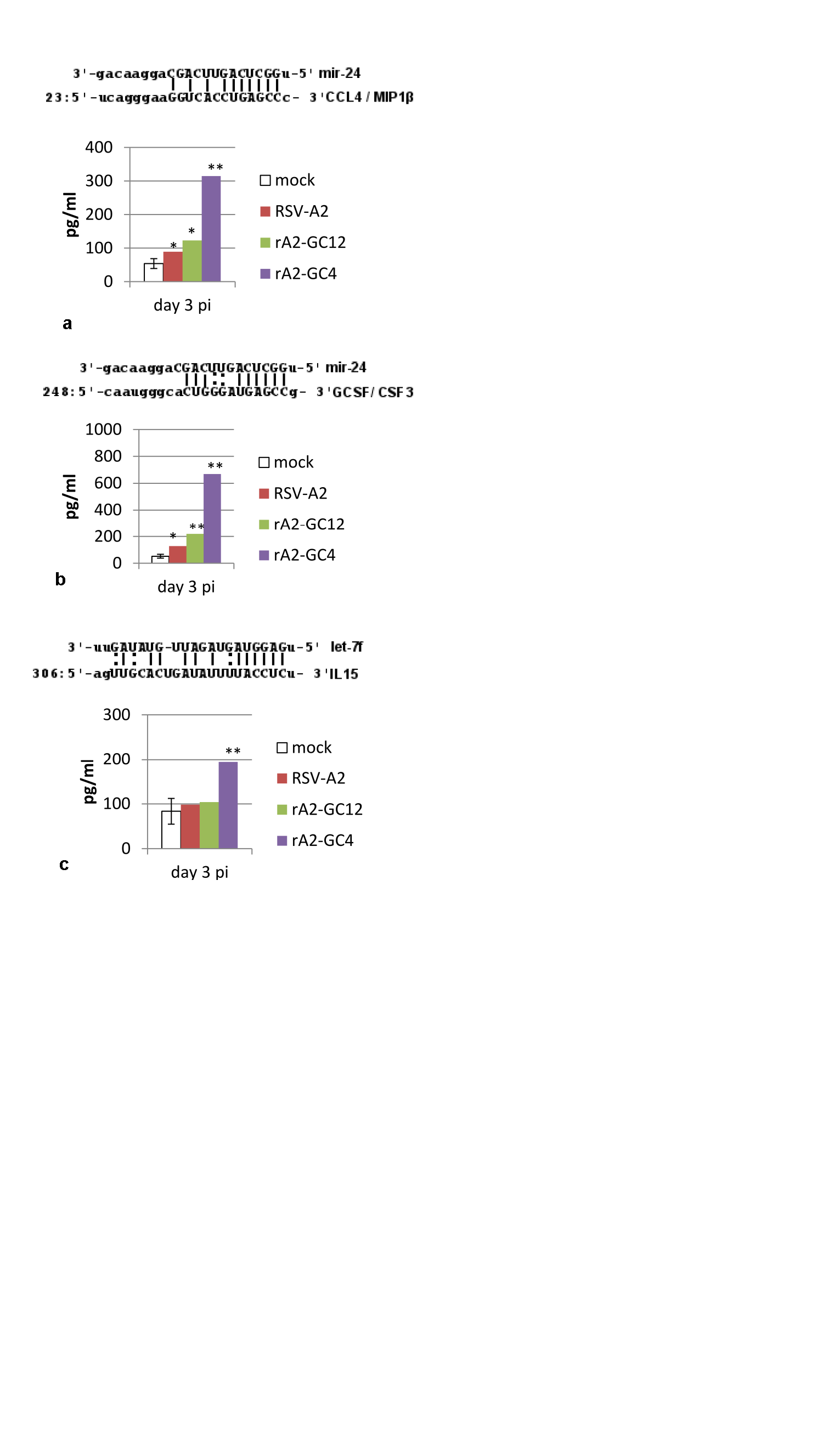


**Figure S1.** Deregulation of miR-24 / let-7f is accompanied with cytokine dysregulation. Protein levels of cytokines CCL4/ MIP-1β (a), GCSF /CSF3 (b) and IL15 (c) were measured in supernatants of polarized Calu3 cells infected with either wild type RSV A2, or rA2-GC12 or rA2-GC4 3d p.i using Luminex assays. Data represent means values of three independent experiments. Alignments of miRNA and the miRNA binding site (MRE) in the 3’UTRs of these genes are shown above expression profiles for each cytokine. * p<0.05 relative to mock, ** p<0.05 relative to both mock treated cells and RSV A2.
